# Editorial: Sport and active living: nature challenge vis-à-vis inter-human competition

**DOI:** 10.3389/fspor.2025.1649600

**Published:** 2025-07-15

**Authors:** Robert Alan Stebbins, François Gravelle, Lee Davidson, Sean Gammon

**Affiliations:** ^1^ Department of Sociology, University of Calgary, Calgary, AB, Canada; ^2^ School of Human Kinetics, Faculty of Health Sciences, University of Ottawa, Ottawa, ON, Canada; ^3^ Victoria University of Wellington, Wellington, New Zealand; ^4^ Business School, University of Lancashire, Preston, United Kingdom

**Keywords:** sport, active living, canyoning, nature challenge, nature challenge activity

**Editorial on the Research Topic**
Sport and active living: nature challenge vis-à-vis inter-human competition

Much of the theory and sociological and social psychological research on sports view this activity in competitive terms; people engage in sport to compete against other participants be they present or historical, in real time or on paper (e.g., most career home runs, largest of a species of fish). Outings in nature regarded as leisure activity constitute a main way in which today's *Homo otiosis* uses his free time. We define nature as any natural setting perceived by users as at most only minimally modified by human beings. Here we define and describe a modern leisure phenomenon called nature challenge activity (NCA), a type of outdoor pursuit that appears to be gaining popularity. The NCA is leisure whose core activity or activities center on meeting a natural test posed by one or more of six elements: (1) air, (2) water, (3) land, (4) animals (including birds and fish), (5) plants, and (6) ice or snow (sometimes both). A main reason for engaging in a particular NCA is to experience participation in its core activities pursued in a natural setting. In other words, while executing these activities, the special (aesthetic) appeal of the natural environment in which this process occurs simultaneously sets the challenge the participant seeks. Note that the six elements of nature are not equally appealing to all people. That is, nature can also be viewed along an aesthetic dimension. That is most of them are attracted only to some of the elements of nature and only to some of the NCAs available there.

By sport we mean inter-human, competitive, physical activity based on a recognized set of rules and possibly related customs. The Shorter Oxford English Dictionary (5th ed.) defines sport similarly, as “an activity involving physical exertion and skill, *esp.* one in which an individual competes against another or others to achieve the best performance”. These definitions include inter-human competition carried out using animals, machines, and the like (e.g., racing with dogs, snowmobiles), providing it requires physical exertion and skill. Finally, chance is not an essential element in a sport, even though chance conditions may influence the outcome of a match. The following exemplify the NCAs:

• Hobbyist collecting of natural objects (shells, leaves, rocks, etc.)
• Orienteering
• Back-country skiing, snowboarding
• Caving, canyoning
• Mushrooming (gathering edibles or scientific specimens)
• Bird watching
• Amateur ornithology, astronomy, botany, entomology, meteorology, mineralogy – the natural challenge is to find new phenomena, rare but known phenomena, predict weather, and so on.
• Search and rescue, maintaining hiking and cross-country ski trails, volunteering as an NCA
• Sailing
• Wilderness camping
• Trapping

Be that as it may, most activity participants seem to prefer one of the other two kinds of hobbies in this subtype: nature activities and corporeal activities in nature.

## Nature activities

This extremely diverse set of interests is pursued in the outdoors. Sorted here into the categories of nature appreciation, nature challenge, and nature exploitation, most are enjoyed most of the time away from towns and cities. Still, within the natural areas in the towns and cities, we may be able to fish, watch birds, cross-country ski, and fly model airplanes, to mention a few possibilities.

## Nature appreciation

At the center of the nature appreciation activities lies the awe-inspiring natural environment in which they take place. Seeing, hearing, smelling, and feeling the surroundings — “getting out in nature” — add up to a powerful reason for doing one or more of the following:

• hiking
• horse riding
• back packing/wilderness camping
• spelunking (cave exploration)
• bird-watching
• canoeing/kayaking
• scuba diving/snorkeling
• snowshoeing
• snowmobiling

Another important reason for pursuing these activities is to learn and express the skills and knowledge needed to find fulfillment in them. At this level they are serious leisure.

## Nature's challenges

A nature challenge activity (NCA) is a leisure pursuit whose core activity or activities centre on meeting a test posed by the surrounding natural environment. As have pointed out elsewhere, considerable nature appreciation is also possible in these activities, though at times the challenges are so stiff that they concentrate the mind more or less exclusively on trying to meet them. These activities include:

• ballooning
• flying
• gliding
• wave surfing
• alpine skiing
• snowboarding
• scuba diving
• cross-country skiing
• sailing (with sail/engine)
• parachuting and skydiving
• hang gliding
• mountain climbing
• white water canoeing and kayaking
• dirt (trail) bike riding (non-competitive)

Thus, an accomplished cross-country skier can savor the beauty of the snow-covered trees and partially frozen streams near trails set on moderate terrain. But then, a steep descent with a sharp turn in the middle suddenly diverts all attention to skiing technique.

## Nature exploitation

In these hobbies, if all goes well, participants come away from their sessions in nature with some of its “yield,” as experienced in among others:

• fishing
• hunting
• trapping
• mushroom gathering

Yet, the fishers, hunters, and others do appreciate nature as well, though less when they are in flow with a fish on the line or a deer in their sights.

A number of familiar outdoor activities are excluded from these three lists, primarily because they are casual rather than serious leisure (e.g., camping in parks, berry picking, beachcombing). Furthermore, some of the activities just discussed, including sailing, alpine skiing, and cross-country skiing, are sometimes also pursued as exploitation.

The four articles in this issue of *Frontiers in Sports and Active Living* give substance to various aspects of this Editorial. The article by Gravelle et al. on lifelong involvement in Olympic weightlifting, which is certainly challenging albeit accomplished indoors as is indoor wall climbing as a substitute for technical outdoor mountain climbing. Sirost et al. write about a variety of wind driven serious challenges in water, on beach, and in air. The next article is that of Afriandy Rangkuti et al., which reports a study of developing sports tourism in Indonesia. This issue of *Frontiers* closes with another study related to nature; namely, one of the flow experience and environmental behaviors by Akçakese et al.

